# Cardiac progenitors cells for vascular repair

**DOI:** 10.18632/aging.101840

**Published:** 2019-02-24

**Authors:** Diego Herrero, Antonio Bernad

**Affiliations:** 1 Cardiac Stem Cell Group, Department of Immunology & Oncology, National Center for Biotechnology, 28049 Madrid, Spain

**Keywords:** heart, progenitor, Bmi1, neovascularization, infarction

The heart is the first functional organ to develop, and cardiomyocytes (cardiac muscle cells) are the essential- and specific-cell type that supports its function during the entire lifespan, being highly resistant to cell damage and aging. Cardiomyocytes occupy ≈ 80% of the volume of mammalian heart, however, they are relatively few in total number compared with non-myocyte cells (endothelial cells, smooth muscle cells, fibroblasts; ≈ 70% of total cardiac cells) [1]. Both myocytes and non-myocytes respond to physiological and pathological insults and their maladaptive responses are linked with the pathogenesis of the cardiac tissue.

During the last decade, various studies have identified cardiac progenitor-like cells, including immature cardiomyocytes, that contribute to the low cardiomyocyte turnover (< 2% per year), decreasing their contribution in an age-dependent manner. While cardiac regenerative response is effective in embryo and neonatal period (until 7th day after birth), the regeneration is particularly limited from adolescence where ischemic injury lead to the formation of a fibrotic scar and to the reduction in the heart's pumping capacity in mice (reviewed in [2]).

The majority of the research in the field of cardiac regeneration have been focused on limiting the death of cardiomyocytes and on looking for the source of *de novo* cardiomyocytes. Despite the remarkable exchange rate of endothelial cells in adult heart (>15% per year) and their essential functions, the cellular source of mature endothelial cells in homeostasis and after injury is not characterized. He et al*.* traced adult cardiac endothelial cells using a genetic lineage-tracing strategy based on fluorochrome expression. Lineage dilution analysis based on the presence or absence of fluorochrome-positive endothelial cells showed that preexisting endothelium (or endothelial-related cells) mediated the neovascularization after infarction [3]. It is important to note that these lineage-tracing experiments were realized based on one endothelial-related gene (*cd31, cdh5*) whose expression is not only restricted to mature endothelial cells. Therefore, it would be possible that cardiac progenitors which express endothelial-related genes could mediate the neovascularization response to myocardial infarction.

In this context, several studies have found cardiac progenitor cells located along cardiac vasculature such as Sca1^+^ adventitial and Gli1^+^ fibroblast-progenitor cells [4]. We recently identified a cell population with adult cardiac progenitor characteristics that expresses high level of BMI1 (B lymphoma Mo-MLV insertion region 1 homolog) protein [5]. Polycomb complex BMI1 expression is widely linked to the regenerative capacity of adult tissues and identifies cells with progenitor-related characteristics in several tissues. In the mammalian heart, Bmi1^+^ cardiac progenitors are a heterogeneous cell population (≈1x10^5^ cells/ adult heart) located in perivascular position that contributes to the three main cardiac cell lineages in homeostasis, increasing this contribution after several types of injury [6,7]. Surprisingly, genetic ablation of Bmi1^+^ cardiac progenitors in homeostasis did not provoke cardiac dysfunction or mice death, probably due to cell plasticity events as showed in other tissue adult stem cells. The role of Bmi1^+^ progenitor cells, however, became essential in the neovascularization process after myocardial infarction (from 15-days to 2-months), contributing up to 20% of new endothelial cells. Genetic ablation of Bmi1^+^ progenitors before infarction confirmed that they are necessary for cardiac physiological remodeling and their absence led to cardiac dysfunction and increased mice death [8].

**Figure 1:**
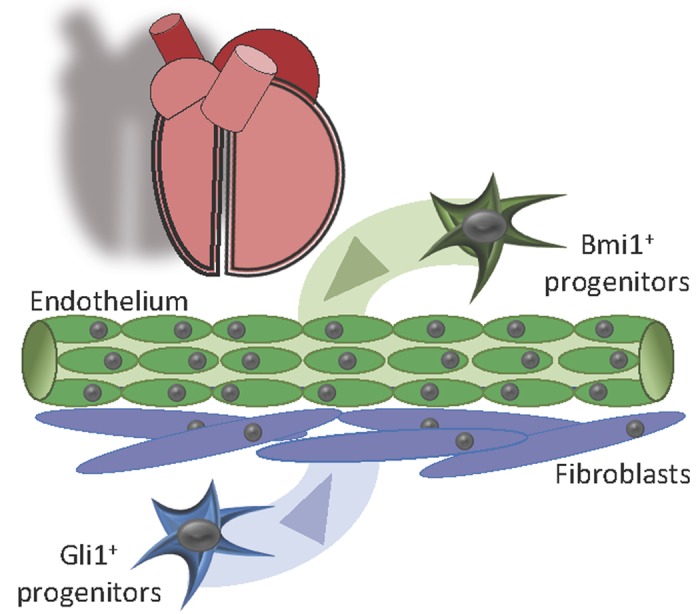
**Lineage-specific cardiac progenitor cells.** Overall, our results suggest that the aging and pathological damage of Bmi1^+^ cardiac progenitors could play an important role in heart aging, exacerbating certain pathological responses. Stimulation of endogenous Bmi1^+^ cardiac progenitor cells in the infarcted myocardium would help counteract the pathological remodeling by sustaining injury-induced neovascularization.

Until a few years ago, the quest of cardiac-resident stem cells was restricted to multipotent, clonogenic cells which should be necessary and sufficient to regenerate the heart efficiently, as showed for hematopoietic or intestinal stem cells. The latest studies suggest the presence of lineage-specific progenitor cells with a key role in heart homeostasis (Figure 1) [4,8].
